# Shock Simulation Day: Medical Decision-Making and Communication Skills for Managing a Hypotensive Adult in a Rapid Response

**DOI:** 10.15766/mep_2374-8265.11430

**Published:** 2024-08-16

**Authors:** A. Vincent Raikhel, Alexandra C. Collis, David Carlbom

**Affiliations:** 1 Clinical Instructor, Division of General Internal Medicine, University of Washington School of Medicine and Veteran Affairs Puget Sound Healthcare System; 2 Clinical Assistant Professor, Division of General Internal Medicine and Division of Healthcare Simulation Science, University of Washington School of Medicine; 3 Associate Professor, Division of Pulmonary, Critical Care, and Sleep Medicine, University of Washington Medical Center

**Keywords:** Rapid Response, Shock, Internal Medicine, Simulation

## Abstract

**Introduction:**

Shock is a life-threatening condition amongst hospitalized patients and requires urgent management to avoid mortality. Early exposure is vital for educational and patient safety purposes.

**Methods:**

We developed a 90-minute shock day session that provided internal medicine interns with a cognitive framework for the initial diagnosis and management of shock, which they applied to two simulations. The first simulation involved a patient with septic shock, and the second involved a patient with cardiogenic shock. Critical action checklists were used to assess learners and guide structured debriefs after each simulation. Medical decision-making and communication frameworks were presented through a presession video and a chalk talk. The curriculum was evaluated using pre- and postintervention surveys to assess knowledge and confidence.

**Results:**

Forty-eight interns participated in the session in 2022 and 2023. We observed an increase in the percentage of learners correctly answering a knowledge-based question regarding the amount of fluid administered to a patient in septic shock (pre: 33%, post: 62%, *p* < .01), as well as increases in learner-reported confidence in leading a rapid response (pre: 9%, post: 62%) and in managing undifferentiated shock (pre: 13%, post: 56%), septic shock (pre: 20%, post: 83%), cardiogenic shock (pre: 2%, post: 54%), hemorrhagic shock (pre: 20%, post: 73%), and anaphylactic shock (pre: 22%, post: 54%, all *p*s < .01).

**Discussion:**

Employing a variety of pedagogical methods, we demonstrated that intern knowledge and confidence regarding the management of a hypotensive patient during a rapid response can be increased through participation in our curriculum.

## Educational Objectives

By the end of this activity, learners will be able to:

1. Apply a framework for determining the etiology of a patient in physiologic shock.
2. Describe initial diagnostic tests required to determine the etiology and severity of a patient in physiologic shock.
3. Describe time-sensitive therapeutic tasks needed to stabilize a patient who is in physiologic shock.
4. Demonstrate rapid response teamwork skills of task delegation and closed-loop communication.

## Introduction

Physiologic shock is a life-threatening condition that results from inadequate oxygen delivery to meet the metabolic demand of tissues; it is often accompanied by hypotension.^1^ Shock is a common clinical condition amongst hospitalized patients, within both the acute care and ICU settings, and requires urgent diagnosis and management in order to avoid significant morbidity and mortality.^2,3^ Shock can be classified by four primary categories: hypovolemic, cardiogenic, distributive, and obstructive.^1^ Determining which type of shock is driving a patient's decompensation is a critical and challenging skill required for independent medical practice in the hospital setting. Due to shock's clinical prevalence and severity, early exposure to caring for a patient with shock during GME training is vital for educational and patient safety purposes.

Patients who acutely decompensate while hospitalized on acute care services, due to physiologic shock or other causes, are often supported by a rapid response team (RRT).^4^ RRTs are required by the Joint Commission and have been integrated into the clinical work of most hospitals within the United States.^5^ RRTs vary in composition but can include a responding physician, STAT nurse, respiratory therapist, and bedside nurse. Within this team, a responding physician typically assumes the role of the RRT leader. In the academic setting, the role of RRT leader is often played by a resident physician. Early in their training, resident physicians can feel ill equipped to take on the role of rapid response leader, which requires leadership and communication skills in addition to medical decision-making. Additionally, it can be challenging to give novice providers enough autonomy during real-life rapid response events to adequately foster learning while also prioritizing patient safety.

Simulation-based education is a powerful pedagogical tool for developing practical skills, including communication, teamwork, and leadership. Using rapid response simulations, early learners can practice these skills in a lifelike, clinically dynamic environment without compromising patient care. Simulation-based learning has the added benefit of improving learners’ situation-specific self-confidence and thereby engaging their self-efficacy. Because of this, simulation has become increasingly integrated into GME curricula across many specialties.^6–8^ While simulations of patients in various types of shock have been previously published,^9^ there are no publications describing a single, comprehensive session that provide learners with a framework for how to approach all four categories of shock within the rapid response environment. Our shock day session provides learners with a cognitive framework for the initial diagnosis and management of shock, which they then apply to two high-fidelity simulations to reinforce their learning. This allowed early resident physicians to obtain practical tools that can be employed in their clinical practice, increase their knowledge about how to manage shock, and enhance their confidence in leading a rapid response.

## Methods

### Development

The session has been offered annually since 2022 as part of the University of Washington School of Medicine internal medicine residency curriculum, and survey data were collected from the 2022 and 2023 sessions. The session was developed by three physicians: two hospitalists and one pulmonary and critical care (PCC) physician. The two hospitalists codirected the intern rapid response simulation curriculum for the internal medicine residency, which involved the development, assessment, and implementation of multiple rapid response simulations across a three-hospital training system. One hospitalist was the director of the internal medicine residency clinician educator pathway and a member of the internal medicine residency core faculty. The other hospitalist was the director of the transition-to-residency course and a member of the internal medicine residency core faculty. The PCC physician was a nationally recognized expert in resuscitation and served on local and national committees for these purposes. The session was implemented at the three hospitals serving as training sites for the internal medicine residency. These three hospitals consisted of an academic tertiary care center, a tertiary care county safety-net hospital affiliated with our university, and a tertiary care Veterans Affairs hospital affiliated with our university. Internal medicine interns at our institution rotated at all three sites throughout their PGY 1 year. Each session was typically attended by six to 10 learners and lasted approximately 90 minutes. The curriculum and all data collected received approval from the University of Washington Institutional Review Board (STUDY00002261).

Our learners consisted of all interns rotating on acute care medicine, medical intensive care, cardiology, and hematology oncology admitting services, as well as inpatient consulting services at the three hospitals within our university's training program. Learner specialties consisted primarily of internal medicine but also included anesthesia, psychiatry, physical medicine and rehabilitation, and family medicine interns who were on an inpatient internal medicine rotation. Participation was required as part of a broader yearlong intern curriculum. The curriculum consisted of four main sessions in the following order: altered mental status, shock, respiratory distress, and arrhythmia. All sessions were structured in a similar fashion. Senior team members were asked to cover patient care clinical duties for the session to support intern attendance.

The primary goal of the curriculum was to increase intern confidence and knowledge in the initial management of an acute care patient who had developed undifferentiated hypotension. The curriculum included a framework for differentiating the four primary categories of shock: hypovolemic, cardiogenic, distributive, and obstructive. The curriculum also offered an opportunity for the development of communication, teamwork, and leadership skills necessary for the effective management of a rapid response.

Prior to participation in the session, learners were instructed to watch a video module that we developed demonstrating an RRT managing a patient with hemorrhagic shock due to a variceal bleed (Appendix A). The video was emailed to learners 1 week before participation along with logistical scheduling and location information for the session. The video demonstrated effective teamwork and communication techniques while also reviewing resuscitation medical decision-making.

The simulation session was structured as shown in Table 1. Each session started with the facilitators introducing the simulation environment and themselves. Learners were provided with guidance on the properties and limitations of working with the high-fidelity manikin. They were encouraged to suspend disbelief to support a more impactful learning experience. During the introduction, facilitators highlighted the importance of psychological safety during the simulation for the learners.^10^

**Table 1.**
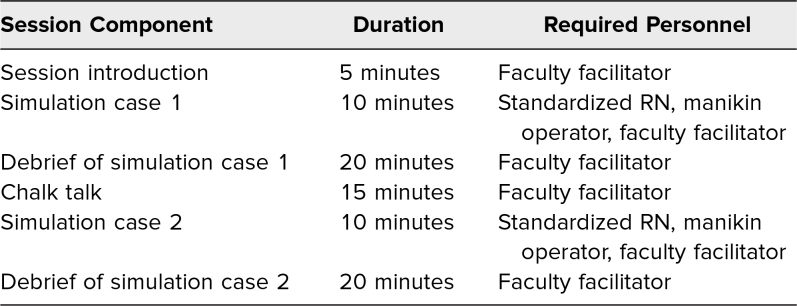
Circular Intervention Component Sequence

At the end of the introduction, the learners were divided into two groups of three to five individuals each. One group was selected to be the active participants in the first simulation. A volunteer from the group was asked to serve in the role of rapid response leader. If a participant did not volunteer, the session facilitator randomly selected a rapid response leader. Active participants who were not the rapid response leader were instructed to play internal medicine interns on a night shift who had responded to assist with a rapid response. They were otherwise not assigned a specific role or responsibility in the simulation. The learners who were not active participants remained inside the room and observed their peers during the first simulation. The observers for the first simulation became active participants in the second simulation. A facilitator observed the simulation from the simulation control room and scored active participants using a critical action checklist (Appendices B and C). Following the simulation, the facilitator led a structured debrief, guided by the completed checklist.

The critical action checklists were developed through a review of literature and local expert opinion regarding hypotension resuscitation and rapid response best practices. Additionally, we ensured that the critical action checklists were consistent with local clinical decision-making tools designed to support the management of patients during a rapid response. The structure of the critical action checklists was adapted from a previously published rapid response performance checklist.^11^

A chalk talk on the management of shock during a rapid response followed the first simulation (Appendix D shows the chalk talk board). A facilitator guide for the chalk talk is provided in Appendix E. The chalk talk was followed by the second simulation and structured debrief.

### Equipment/Environment

The simulations were all performed in dedicated simulation labs designed to mirror resources of an acute care medical/surgical ward.

• Simulated environment: telemetry floor.
• Equipment:
  ○ High-fidelity human patient simulator;
  ○ Monitor for real-time simulated monitoring of the heart rate, blood pressure, oxygen saturation, and temperature;
  ○ Telemetry leads;
  ○ Medication syringes;
  ○ Liter bags of fluids;
  ○ IV tubing and IV poles;
  ○ Nasal canula tubing; and
  ○ Hospital gown.
• Standardized participant: one standardized nurse (an RN) who administered all medications, crystalloid fluids, and blood products, as well as providing imaging and laboratory results and limited medical history.

### Personnel

Two faculty members were present for each session. The faculty included two hospitalists and one PCC physician. The high-fidelity human patient simulator was run with the help of simulation technicians at two of the sites. The simulator was run by a hospitalist at the third site. The nurse was performed by an RN who specialized in simulation training at one of the sites and was performed by a faculty member at the other sites.

### Implementation

Both cases began with the rapid response leader and team presenting to bedside after being called by the bedside nurse. The learners were stationed outside the simulation room before the simulation started. They were handed the patient's sign-out documentation (Appendix F for case 1 and Appendix G for case 2) and allowed to review the patient's data. After 1–2 minutes, the nurse opened the door and asked the learners to enter the room. The two cases were presented in the simulation case files (Appendices H and I). Supplementary data for each case, including electrocardiograms, chest radiographs, and laboratory data for the cases, were available (Appendices J and K). Learners were notified at the beginning of the session that they could utilize their phones during the simulation to access any resources they would use during a real-life event to support their clinical decision-making.

### Debriefing

Immediately after each simulation, a faculty facilitator led a structured debrief. The debrief was divided into two domains: (1) teamwork, communication, and leadership and (2) medical knowledge, reasoning, and application. The standardized debrief (Appendix L) was designed based on PEARLS (Promoting Excellence and Reflective Learning in Simulation).^12^ Each component of the debrief started with a discussion of had what gone well, followed by discussions of what could be improved. During the discussion, the facilitator elicited the perspectives of the standardized nurse, rapid response leader, other active participants, and observing learners. The facilitator used the results of the critical action checklist to guide the discussions in both domains. Elements on the checklist that had been missed during the simulation were reflected to learners as an opportunity for discussion and growth. The debriefing guide (Appendix L) included a structure for the debrief discussion in addition to key teaching points. Critical action checklists for the two simulations were developed based on expert opinion of two hospitalists and one critical care physician and on critical action checklists developed previously in the literature.^11^ Items on the critical action checklists focused on clinically significant and observable elements that supported subsequent discussions during the postsimulation debrief.^12^

### Assessment

Interns were given voluntary pre- and postparticipation surveys (Appendices M and N) to complete. The preparticipation survey was given immediately prior to participation, and the postparticipation survey was given immediately following completion of the final debrief. Surveys were completed on paper. Participants were still allowed to participate in the educational activities if they declined to take the survey. Learners who arrived late for the activity were not given preparticipation surveys as this would have disrupted the flow of the session and inhibited learner participation. Surveys obtained data on learners’ previous experience managing rapid responses, confidence managing various subtypes of shock, and knowledge of managing physiologic shock. Knowledge-based questions consisted of some open-ended and some multiple-choice items. Participants rated their confidence in managing a rapid response and managing different presentations of shock on a 5-point Likert scare (1 = *not at all,* 2 = *not very,* 3 = *neutral,* 4 = *somewhat,* 5 = *extremely*). Confidence-related Likert responses were consolidated into not confident (1 = *not at all,* 2 = *not very,* 3 = *neutral*) and confident (4 = *somewhat,* 5 = *extremely*) and compared using chi-square analysis to determine statistical significance.

## Results

Forty-eight learners participated in the session between 2022 and 2023. Forty-five learners completed the preparticipation survey, and 48 completed the postparticipation survey. All participants (48 of 48, 100%) reported that they thought the session improved their ability to manage a patient in shock, and 93% (45 of 48) reported that the session improved their leadership skills.

Interns reported having participated in zero (two of 45, 4%), one (three of 45, 6%), two to three (19 of 45, 42%), or four or more (21 of 45, 46%) rapid responses during their intern year. We observed an increase in the correct response to the open-ended, knowledge-based question “How much fluid should be administered to a patient who is in septic shock?” (pre: 15 of 45, 33%; post: 30 of 48, 62%; *p* < .01). We did not observe a statistically significant increase in tested knowledge for labs that should be ordered for a patient in septic shock (pre: 24 of 45, 53%; post: 31 of 48, 64%; *p* = .20). There was a statistically significant increase in learner confidence in leading a rapid response (pre: four of 45, 9%; post: 30 of 48, 63%; *p* < .01). Learners reported statistically significant increases in confidence in leading a rapid response and in managing each of the types of shock that were assessed (Table 2). All participants (48 of 48, 100%) reported that they felt the session improved their ability to manage a patient in shock and that they would like to attend more rapid response simulations.

**Table 2.**
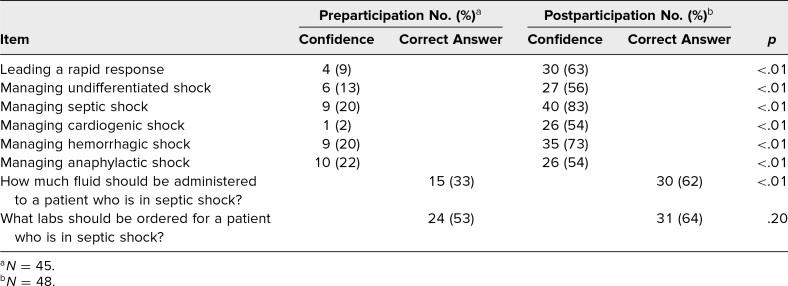
Pre- and Postparticipation Confidence in Managing a Rapid Response and Knowledge of Managing Specific Subtypes of Shock

Participants were also able to provide open-ended feedback for the session. Representative comments included the following: “Excellent Learning opportunity. I really appreciated the focus on team leadership/communication in addition to knowledge” and “This was a great experience, decreases anxiety around rapids and great leads and practice.” Comments also included a request for more frequent sessions—“More sessions would be very helpful!”—and suggestions for how to optimize the timing of the session—“Not on intern switch week! Maybe week 3 or 4 when our rotation flow is better.”

## Discussion

Managing a patient in physiologic shock during a rapid response is a critical skill for in-hospital medical providers and an area of vulnerability for early resident physicians. We have demonstrated that intern knowledge and confidence in managing a hypotensive patient during a rapid response was increased following participation in our shock day curriculum. While previous simulation-based curricula have included shock, our innovation is unique in that it provides a general framework for approaching undifferentiated shock in the context of a rapid response through a variety of educational strategies, with an opportunity to apply this framework in two simulation cases. Utilizing differing strategies, including asynchronous video, interactive simulation, and didactic chalk talk, allows for spaced repetition of the material. It also enables a wide variety of learning styles to be accommodated and supported during the curriculum.

We believe that the combination of teaching techniques utilized in this intervention, when paired proximately, primes participating learners’ self-efficacy, a key component of improving performance and behavior through social learning thoery.^13^ The prework video provides a vicarious experience by showing an example of managing a patient in shock and modeling effective communication, teamwork, and leadership. Participating as an active participant and observer between the two simulations with paired debriefs empowers social persuasion, as learners can receive feedback on their performance of a complex task while also learning from observing peer behaviors. The debriefs also allow for reflection on the emotional state of learners during a simulated high-stress clinical scenario, which may improve self-efficacy by offering insights into the emotional and physiological states learners experience.

The chalk talk provides learners with a clear cognitive framework for differentiating between the four major categories of shock in an acutely decompensating patient and determining how to initiate resuscitation in each case. Following the chalk talk, learners have a second opportunity to apply this framework to a simulated patient. This interleaving of material is designed to reinforce learner application and integration of key teaching points as well as to promote retention.

In their comments, residents noted that this was a helpful session that decreased their anxiety around leading rapid responses in the clinical environment. In particular, residents appreciated being able to practice leadership and communication skills in a safe environment. The critical action checklists offer faculty the ability to provide specific feedback on important diagnostic, therapeutic, and communication elements that may not have been completed or have been completed in a manner that leaves room for improvement during the simulation. This opportunity to reflect on and discuss why specific elements of care may have been suboptimal offers the chance for learner growth and development while reinforcing the session's learning objectives.

Although we did see increases in learners who reported confidence after participation in the session, a sizable number of other learners reported feeling neutral or unconfident in the queried domains. Despite this, 100% (48 of 48) reported that they felt the session improved their ability to manage a patient in shock. We did not see a statistically significant change in the response to the question regarding what labs to order for a patient in septic shock. This may have been partially impacted by the question being open-ended. We scored as correct the responses that included a complete blood count, lactate, blood culture, and urinalysis with culture. Other answers featured other lab combinations that could be relevant for diagnostic reasoning within the context of a rapid response, including arterial blood gas, complete metabolic panel, troponin, and coagulation studies, but missed one of the core sepsis labs. This may indicate that this learning point's clarity could be improved to support learner retention. Given the complex nature of leading a rapid response and diagnosing and managing shock, it is unlikely that a single educational innovation is sufficient to support independent practice. Our session takes place within the context of a broader intern curriculum that includes lectures, case-based discussions, and other simulations. We suggest that other institutions implement this shock day session similarly, providing additional educational scaffolding to support learner growth.

There are limitations to the evaluation of our innovation. The session was implemented within a single residency program, and our observations may not be generalizable to a broader group of learners. Our data were collected using a survey that introduced risk of recall bias, as the survey was completed after participation. The survey data were not collected using anonymized surveys, which increased the risk for social desirability bias. Additionally, our confidence data may not reflect an actual improvement in patient care abilities. Implementation of this session requires a significant amount of time, equipment, and personnel resources that may not be available to all training programs. While a variety of trainees from different specialties participated in the program, the participating cohort did not include learners from any surgical specialties, limiting the diversity of the learning cohort. The skills of leading a rapid response and managing shock are applicable to a wide variety of specialties that practice medicine within the hospital setting. Assessing the impact of this curriculum on a wider variety of medical specialties is an area for future investigation. Furthermore, evaluating learners’ retention of the learning objectives and assessing any integration into clinical practice are areas of interest that would benefit from further investigation.

## Appendices


Rapid Response Variceal Bleed Video.mp4Case 1 Critical Action Checklist.docxCase 2 Critical Action Checklist.docxShock Chalk Talk.docxShock Chalk Talk Instructions.docxCase 1 Patient Sign-out.docxCase 2 Patient Sign-out.docxCase 1 Facilitator Guide.docxCase 2 Facilitator Guide.docxCase 1 Supplemental Data.docxCase 2 Supplemental Data.docxDebrief Guide.docxShock Presimulation Survey.docxShock Postsimulation Survey.docx
*All appendices are peer reviewed as integral parts of the Original Publication.*
