# Supplementary material for: Shock Simulation Day: Medical Decision-Making and Communication Skills for Managing a Hypotensive Adult in a Rapid Response
Source: MedEdPORTAL. 2024 Aug 16;20:11430. doi: 10.15766/mep_2374-8265.11430 (PMC11327352; doi:10.15766/mep_2374-8265.11430)
Supplement: Supplementary file 1 — Rapid Response Variceal Bleed Video.mp4Case 1 Critical Action Checklist.docxCase 2 Critical Action Checklist.docxShock Chalk Talk.docxShock Chalk Talk Instructions.docxCase 1 Patient Sign-out.docxCase 2 Patient Sign-out.docxCase 1 Facilitator Guide.docxCase 2 Facilitator Guide.docxCase 1 Supplemental Data.docxCase 2 Supplemental Data.docxDebrief Guide.docxShock Presimulation Survey.docxShock Postsimulation Survey.docx [file mep_2374-8265.11430-s001.zip › B. Case 1 Critical Action Checklist.docx]

**Appendix B. Instructions:** To be used by faculty facilitator during simulation. Refer to what was completed and not completed on the checklist during the post simulation debrief.

| **Sepsis Critical Action Checklist** | | | | | **Date:**  **Site:**  **Leader:**  **Others:** | | | |
| --- | --- | --- | --- | --- | --- | --- | --- | --- |
| **Evaluator:** | | | | |  |  |  |  |
| **Rapid Response Leadership** | | | | |  |  |  |  |
| 1. Identifies self as leader of RRT | | | | | | | |  |
| 2. Asks for vital signs | | | | | | | |  |
| 3. Stands at foot of bed | | | | | | | |  |
| 4. Assigns roles (or identifies roles) | | | | | | | |  |
| 5. Asks for events leading up to RRT | | | | | | | |  |
| 6. Asks for current medication list | | | | | | | |  |
| 7. Asks for past medical history | | | | | | | |  |
| 8. Asks for recent labs | | | | | | | |  |
| **Initial Assessment Shock** | | | | | | | | |
| 1. Identifies shock | | | | | | | |  |
| 2. Identifies sepsis | | | | | | | |  |
| 3. Obtains PIV | | | | | | | |  |
| **Labs ordered** | | | | | | | | |
| Lactate |  | Blood cultures |  | CBC | |  | BMP/CMP |  |
| **Treatment/drugs** | | | | | | | | |
| 4. Broad spectrum antibiotics (to include Cefepime + Metronidazole, Piperacillin/tazobactam, or Carbapenem +/- Vancomycin) | | | | | | | |  |
| 5. IV fluid bolus or vasopressor (30cc/kg or at least 1L) | | | | | | | |  |
| **Other Comments:** | | | | | | | | |
|  |  |  |  |  |  |  |  |  |
