# Supplementary material for: Shock Simulation Day: Medical Decision-Making and Communication Skills for Managing a Hypotensive Adult in a Rapid Response
Source: MedEdPORTAL. 2024 Aug 16;20:11430. doi: 10.15766/mep_2374-8265.11430 (PMC11327352; doi:10.15766/mep_2374-8265.11430)
Supplement: Supplementary file 1 — Rapid Response Variceal Bleed Video.mp4Case 1 Critical Action Checklist.docxCase 2 Critical Action Checklist.docxShock Chalk Talk.docxShock Chalk Talk Instructions.docxCase 1 Patient Sign-out.docxCase 2 Patient Sign-out.docxCase 1 Facilitator Guide.docxCase 2 Facilitator Guide.docxCase 1 Supplemental Data.docxCase 2 Supplemental Data.docxDebrief Guide.docxShock Presimulation Survey.docxShock Postsimulation Survey.docx [file mep_2374-8265.11430-s001.zip › H. Case 1 Facilitator Guide.docx]

**Appendix H.** Case 1 Facilitator Guide

| **Simulation case title: Shocked to the Core**  **Authors:**  **Vince Raikhel, MD**  **Alexandra Collis, MD**  **David Carlbom, MD** | |
| --- | --- |
| **Patient name: Justin Ranka**  **Patient Age: 59** | |
| **Simulation duration: approximately 10 minutes** | |
| **Brief narrative description of case** | Justin Ranka is a 59-year-old man with a PMH of DMT2, HTN, and Gout who presented to the Emergency Department last night with left lower quadrant abdominal pain and was found to have an obstructing left ureteral stone and hyperglycemia. He underwent lithotripsy with urology earlier today and tolerated the procedure well. He is anticipating discharging home in the morning. |
| **Learning Objectives** | - Demonstrate effective rapid response communication skills. - Develop a differential diagnosis of circulatory shock that includes sepsis. - Take time-sensitive steps to treat sepsis. - Demonstrate understanding of when additional resources/ services are needed for patient evaluation and management. |
| **Page to rapid response leader** | “RRT pt Ranka is hypotensive” |
| **Medications** | Allopurinol 100 mg Daily  Amlodipine 5 mg daily  Insulin glargine 5 units QHS  Tamsulosin 0.4 mg  Oxycodone 5-10 mg q4h  PRN  Senna 17.2 mg daily  Enoxaparin 40 mg QHS |
| **AM Labs** | WBC 8.3 K/uL, Hct 35 %, Plts 315 K/uL  Na 132 mEq/L, K 4.0 mEq/L, Cl 97 mEq/L,  HCO3 15 mEq/L, BUN 32 mg/dL, Cr 1.1 mg/dL,  Glucose 155 mg/dL |
| **Roles for standardized participant(number of individuals who can be in this role: 1)** | Covering RN (1) |
| **Roles for learners (roles should be preassigned to learners before the simulation begins).** | Primary Senior Resident – rapid response team leader (1)  Interns responding to the rapid response (3-5) |
| **Critical Actions** | - Identify self upon entering the room and assign roles. - Perform focused physical exam. - Articulates a differential diagnosis including sepsis. - Assess patient's vascular access. - Treat hypotension with IVF and/or vasopressors. - Start broad spectrum antibiotics. - If simulation goes to state 3: recognize need for intubation. |
| **Learner Preparation** | - 10 minute video |
| **Manikin setup on leader arrival** | - Monitor: patient is not attached to monitor (monitor off) - Patient is in bed at 45 degrees. - Blood pressure cuff is not applied to patient but is visible in room. - Pulse oximeter is not attached to patient/continuous O2 monitoring not set up - Pulse: patient has an regular tachycardic pulse with rate in ~130s - Access: one 20g PIV present in R AC - Exam: eyes are closed |
| **Materials Needed** | - Manikin - Blood pressure cuff - Peripheral IV - IV Fluids - Antibiotics - Vasopressors |
| **Timing of medication arrival.** | - IV Fluids should be provided to the medical team immediately upon request. - Antibiotics and vasopressors should be provided 1-2 minutes after request. - Any other requested medications should be administered 1-2 minutes after request. |
| **Timing of new data.** | - Laboratory data should be provided to the medical team 2-3 minutes after requested. - ECG should be provided at the time it is requested. - CXR should be provided 1-2 minutes after requested. - Any requested culture data does not result during simulation. - Ultrasound is out of service and cannot be utilized during this simulation. |

| **Physical Examination** | |
| --- | --- |
| **INITIAL VITAL SIGNS** | T 38.2; HR 124 BP 82/65; RR 22; SpO2 96% on room air |
| **General** | Somnolent but responsive to verbal stimuli |
| **Lungs** | Increased RR, normal respiratory depth, clear breath sounds bilaterally. |
| **Cardiovascular** | Tachycardic, regular rhythm, no extra heart sounds |
| **Abdomen** | Flat, non-tender, no rebound or guarding. |
| **Neurological** | somnolent, responsive to verbal stimuli, AOx2, no focal deficits |
| **Skin** | Warm, no rashes |
| **Psychiatric** | Normal affect |

| **Case Stage/ Time Point** | **Case Details** | **Additional Information** |
| --- | --- | --- |
| State 1: Learner enters room and begins care of patient. | - Vital signs T 38.2; HR 124 BP 82/65; RR 22; SpO2 96% on room air - BP increases to 90s/40s for 1-2 minutes after fluid bolus. | **Learner Actions:**   - Learner introduces themselves, assigns team roles. - Learner performs focused physical exam. - Lowers head of bed. - Treat hypotension with IVF Bolus. - Articulate a differential diagnosis including sepsis. - Obtain relevant diagnostic studies.   **Trigger Points:**   - If learners treat hypotension proceed to state 2. - If learners do not treat hypotension proceed to state 3 after 5 minutes |
| State 2: Refractory hypotension and increasing somnolence. | - HR 110-120, O2 saturation 94-98%, RR 24-30 - BP increases to 90s/40s for 1-2 minutes after fluid bolus before returning to 80s/40s. Patient is now unresponsive to any verbal stimuli, minimally responsive noxious tactile stimuli. - BP increases to 104/62 if vasopressors are started, BP remains in 100/60s. Patient is minimally responsive to tactile stimuli. - BP increases to 121/70 if fluid bolus and vasopressors are started. Patient is minimally responsive to verbal stimuli. | **Learner Actions:**   - Treat hypotension with IVF Bolus and/or start vasopressors. - Start broad spectrum antibiotics. - Draw blood and urine cultures. - Discuss/ assess IV access. - Call ICU for evaluation and management   **Trigger Points:**   - If learners do not treat hypotension with combination of IVF and vasopressors proceed to state 3 after 5 minutes - The case ends after 10 minutes. |
| State 3: Patient obtunded and hypoxic | - Vital signs: BP 60s/40s, HR 120-130s, O2 saturation 85-89%, RR 24-30 - Patient is now unresponsive to verbal or tactile stimuli. | **Learner Actions:**  **Continue resuscitation with**   - Apply oxygen delivery device. - Call anesthesia for intubation   **Trigger Points:**   - The case ends after 10 minutes. |
| **Case End** |  | **Case End**   - After 10 minutes   **OR**  If the leaner has completed the following tasks:   - Discussed a differential diagnosis for shock including sepsis. - Initiated IVF bolus and vasopressors. - Obtained relevant diagnostic data including cultures, lactate, CXR. - Initiated broad spectrum antibiotics. - Called the MICU for assessment and transfer. |
