# Supplementary material for: Shock Simulation Day: Medical Decision-Making and Communication Skills for Managing a Hypotensive Adult in a Rapid Response
Source: MedEdPORTAL. 2024 Aug 16;20:11430. doi: 10.15766/mep_2374-8265.11430 (PMC11327352; doi:10.15766/mep_2374-8265.11430)
Supplement: Supplementary file 1 — Rapid Response Variceal Bleed Video.mp4Case 1 Critical Action Checklist.docxCase 2 Critical Action Checklist.docxShock Chalk Talk.docxShock Chalk Talk Instructions.docxCase 1 Patient Sign-out.docxCase 2 Patient Sign-out.docxCase 1 Facilitator Guide.docxCase 2 Facilitator Guide.docxCase 1 Supplemental Data.docxCase 2 Supplemental Data.docxDebrief Guide.docxShock Presimulation Survey.docxShock Postsimulation Survey.docx [file mep_2374-8265.11430-s001.zip › I. Case 2 Facilitator Guide.docx]

**Appendix I.** Case 2 Facilitator Guide

| **Simulation case title: The shock of a lifetime.**  **Authors:**  **A. Vincent Raikhel, MD**  **Alexandra Collis, MD**  **David Carlbom, MD** | |
| --- | --- |
| **Patient name: Deborah Black**  **Patient Age: 72** | |
| **Simulation duration: approximately 10 minutes** | |
|  | The case begins with learners being called to the patient's bed due to a rapid response for tachycardia and abdominal discomfort. On initial examination the leaners will find that the patient is tachycardiac with mildly depressed blood pressure. The patient reports that they are experiencing ‘heartburn.’ It is critical that the leaners obtain a focused review of systems as the patient will report that they are also experiencing ‘chest pressure’ and ‘shortness of breath’ if asked. The patient then becomes increasingly lightheaded and hypotensive. Learners must recognize that the patient is going into shock and have a high suspicion for a cardiac etiology for the shock. An electrocardiogram will show a right sided myocardial infarction which is the etiology of her hemodynamic instability. Learners should treat the patient's hypotension with IV fluid. Learners should treat the patient for a STEMI with a heparin ggt, ASA, and Plavix load, in addition to calling cardiology urgently. If learners treat the patient with nitroglycerin or beta blockers the patient will become increasingly unstable. At the completion of the scenario, a debriefing session is performed that includes discussion of the team's communication and teamwork dynamics, in addition to a review of the medical decision making. |
| **Learning Objectives** | **By the end of the simulation, learners will be able to:**   1. Demonstrate closed loop communication during a rapid response. 2. Recognize cardiogenic shock due to a right sided STEMI as a cause of an unstable patient who is hypotensive. 3. Formulate an initial plan for achieving hemodynamic stability in a hypotensive patient due to a STEMI. 4. Formulate an initial plan for management of a patient who is having a STEMI. 5. Determine what medical teams need to be involved in the care of an unstable, hypotensive patient. |
| **Patient HPI** | Deborah Black is a 72-year-old woman with a PMH of DMT2, HTN, HLD, and paroxysmal atrial fibrillation who presented to the Emergency Department two days ago after a ground level fall. Her work up has been negative, and her fall is currently thought to have been mechanical in nature. She will be discharged to a SNF in the morning. Nursing calls RRT and team is asked to evaluate for tachycardia and abdominal discomfort. |
| **Page to rapid response leader** | “RRT pt Black Tachycardic and having abdominal pain” |
| **Medications** | Metformin 1000 mg daily  Lisinopril 20 mg daily  Atorvastatin 40 mg daily  Metoprolol succinate 75 mg daily  Apixaban 5 mg BID  Melatonin 3 mg daily  Cholecalciferol 600 units daily  Acetaminophen 650 q6h PRN  Senna 17.2 mg daily  Polyethylene glycol 17 grams daily PRN |
| **AM Labs** | WBC 5.1 K/uL, Hct 31 %, Plts 273 K/uL  Na 136 mEq/L, K 3.7 mEq/L, Cl 99 mEq/L,HCO3 19 mEq/L, BUN 21 mg/dL, Cr 0.7 mg/dL, Glucose 172 mg/dL |
| **Roles for standardized participant(number of individuals who can be in this role: 1)** | Covering RN (1) |
| **Roles for learners (roles should be preassigned to learners before the simulation begins).** | Primary Senior Resident – rapid response team leader (1)  Interns responding to the rapid response (3-5) |
| **Critical Actions** | - Identify self upon entering the room and assign roles. - Perform focused physical exam. - Requests that an ECG be obtained - Correctly interprets the ECG. - Articulates a differential diagnosis including STEMI. - Treat hypotension with IVF - Call Cardiology - Order ASA and heparin ggt. - Obtain diagnostic studies including (but not limited to) CBC, BMP/CMP, lactate, blood and/or urine cultures, troponin, PT/INR, CXR, ECG. - If simulation goes to state 4: call code blue and start chest compressions. |
| **Learner Preparation** | - 10-minute rapid response shock video |
| **Manikin setup on leader arrival** | - Monitor: patient is not attached to monitor (monitor off) - Patient is in bed at 45 degrees. - Blood pressure cuff is not applied to patient but is visible in room. - Pulse oximeter is not attached to patient/continuous O2 monitoring not set up - Pulse: patient has an regular tachycardic pulse with rate in ~110s - Access: one 20g PIV present in R AC |
| **Materials Needed** | - Manikin - Blood pressure cuff - Peripheral IV - IV Fluids - ASA - Plavix - Heparin ggt |
| **Timing of medication arrival.** | - IV Fluids should be provided to the medical team immediately upon request. - Heparin, ASA, and Plavix should be provided 1-2 minutes after request. - Any other requested medications should be administered 1-2 minutes after request. |
| **Timing of new data.** | - Laboratory data should be provided to the medical team 2-3 minutes after requested. - ECG should be provided at the time it is requested. - CXR should be provided 1-2 minutes after requested. - Any requested culture data does not result during simulation. - Ultrasound is out of service and cannot be utilized during this simulation. |

| **Physical Examination** | |
| --- | --- |
| **INITIAL VITAL SIGNS** | T 37.4; HR 112 BP 92/65; RR 24; SpO2 96% on room air |
| **General** | Anxious, no acute distress |
| **HEENT** | NA |
| **Lungs** | Increased RR, normal respiratory depth, clear breath sounds bilaterally. |
| **Cardiovascular** | Tachycardic, regular rhythm, no extra heart sounds |
| **Abdomen** | Flat, non-tender, no rebound or guarding. |
| **Neurological** | Awake, alert, AOx3, no focal deficits |
| **Skin** | No rashes (not sure how to do this with manikin, but worth having someone note extremities are cold) |
| **GU** | NA |
| **Psychiatric** | Normal affect |

| **Case Stage/ Time Point** | **Case Details** | **Additional Information** |
| --- | --- | --- |
| **State 0: Learners waiting outside simulation room.** | **“RRT pt is hypotensive and reporting abdominal pain, please come to bedside"** | **Trigger Point:**   - **Learners enter simulation room upon receiving page.** |
| **State 1:** Learner enters room and begins care of patient. | - Vital signs T 37.4; HR 112 BP 92/65; RR 24; SpO2 96% on room air - Patient is responsive to verbal stimuli. She endorses feeling “funny, like I have really bad heartburn.” - If asked specifically patient will endorse +slight chest pressure, +SOB, +nausea - If asked, the patient will deny chest pain, radiation, change in discomfort with movement or respiration. | **Learner Actions:**   - Learners introduce themselves, assign team roles. - Learner performs focused interview. - Learner performs focused physical exam. - Obtain and interpret ECG. - Articulate a differential diagnosis concerning myocardial infarction.   **Trigger Points:**   - If learners do not obtain ECG within 5 minutes proceed to state 2. - If ECG is obtained, proceed to state 2 about 30 seconds after ECG is handed to team. - If learners administer nitroglycerin or beta blockers at any time proceed to state 3. |
| **State 2:** Hypotension from right ventricle infarction. | - HR 110-120, BP 82/50, O2 saturation 94-98%, RR 24-30 - Pt reports feeling lightheaded but is responsive to verbal stimuli. - BP increased to 110s/70s if IVF Bolus is administered. | **Learner Actions:**   - Treat hypotension with IVF Bolus. - Recognize possible right ventricular STEMI - obtain right sided ECG. - Ask for recent labs and obtain fresh labs (trop, PT/INT, CBC, BMP, lactate) - Call cardiology - Lower head of bed - Order ASA 324 mg, heparin ggt. - Learner can order Plavix load, or plan to discuss Plavix with cardiology.   **Trigger Points:**   - If learners do not treat hypotension with IVF case remains in State 2 without improvement in BP. - If learners treat hypotension with IVF case remains in STATE 2 with improvement of BP to 110s/70s. - If learners administer nitroglycerin or beta blockers at any time proceed to state 3. |
| **State 3: Post** nitroglycerin hypotension | - Vital signs: BP 60s/40s, HR 120-130s, O2 saturation 90-92%, RR 24-30 - Patient is increasingly confused but responsive to verbal and tactile stimuli. - If IVF bolus is administered BP increases to 80s/50s | **Learner Actions**   - Treat hypotension with IVF. - Apply oxygen delivery device. - Discuss the need for MICU/CCU evaluation for transfer.   **Trigger Points:**   - If learner administers IVF return to state 2. - If learners administer more nitroglycerin or beta blocker at any time in State 3 proceed to state 4. |
| **State 4:** Post multiple doses of nitroglycerin resulting in Ventricular Fibrillation cardiac arrest. | - Vital signs: BP declines further, and patient goes into ventricular fibrillation cardiac arrest. - Patient is completely unresponsive. | **Learner Actions**   - Call code blue - Begin chest compressions.   **Trigger Points:**   - End case when chest compressions begin. |
| **Case End** |  | **Case End**   - After 10 minutes   **OR**   - The patient has gone into cardiac arrest and chest compression have begun.   **OR**  If the leaner has completed the following tasks:   - Discussed a differential diagnosis for hypotension including cardiogenic component. - Initiated IVF bolus. - Ordered ASA load and heparin ggt. - Obtained standard and right sided ECG. - Called cardiology for assessment. - Discussed transfer of the patient to an ICU |
