# Supplementary material for: Shock Simulation Day: Medical Decision-Making and Communication Skills for Managing a Hypotensive Adult in a Rapid Response
Source: MedEdPORTAL. 2024 Aug 16;20:11430. doi: 10.15766/mep_2374-8265.11430 (PMC11327352; doi:10.15766/mep_2374-8265.11430)
Supplement: Supplementary file 1 — Rapid Response Variceal Bleed Video.mp4Case 1 Critical Action Checklist.docxCase 2 Critical Action Checklist.docxShock Chalk Talk.docxShock Chalk Talk Instructions.docxCase 1 Patient Sign-out.docxCase 2 Patient Sign-out.docxCase 1 Facilitator Guide.docxCase 2 Facilitator Guide.docxCase 1 Supplemental Data.docxCase 2 Supplemental Data.docxDebrief Guide.docxShock Presimulation Survey.docxShock Postsimulation Survey.docx [file mep_2374-8265.11430-s001.zip › J. Case 1 Supplemental Data.docx]

**Appendix J Instructions:** Please pace return of labs, imaging, and diagnostics according to when ordered in the scenario (e.g. if ordered early, delay a bit to encourage interventions in the down time and, if ordered late, may return more rapidly to expedite the scenario completion).

| **NEW DIAGNOSTIC DATA (return IF requested ordered)** |  |
| --- | --- |
| **Point-of-care glucose** | 211 mg/dL |
| **Complete blood count** | WBC 17.4 K/uL, Hct 36 %, Plts 512 K/uL |
| **Basic metabolic panel** | Na 129 mEq/L, K 4.1 mEq/L, Cl 96 mEq/L, HCO3 13 mEq/L, BUN 42 mg/dL, Cr 1.6 mg/dL, Ca 9.6 mg/dL, Mg 1.8 mg/dL |
| **Liver function tests** | AST 81 U/L, ALT 92 U/L, Alkaline Phosphatase 103 U/L, TBili 0.4 mg/dL |
| **Arterial Blood Gas** | pH 7.21, pCO2 25 mmHg, pO2 85 mmHg, HCO3 11 mEq/L |
| **Lactate** | 3.2 mmol/L |
| **Troponin** | 0.008 ng/mL |
| **Coagulation studies** | PTT 33.8 secs, PT 12.7 secs, INR 1.2 |

**Chest radiograph**


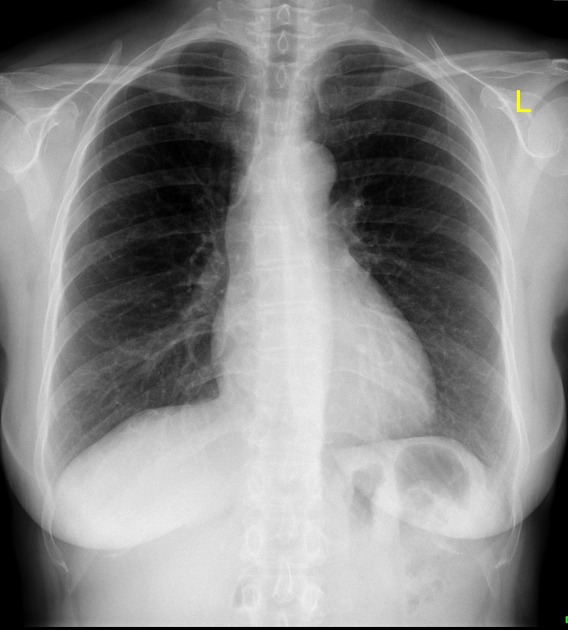


Case courtesy of Henry Knipe, Case courtesy of Henry Knipe, Radiopaedia.org, rID: 3152

Image retrieved from <https://radiopaedia.org/cases/normal-chest-x-ray-1?lang=us> on 3/8/2024.

**Electrocardiogram**


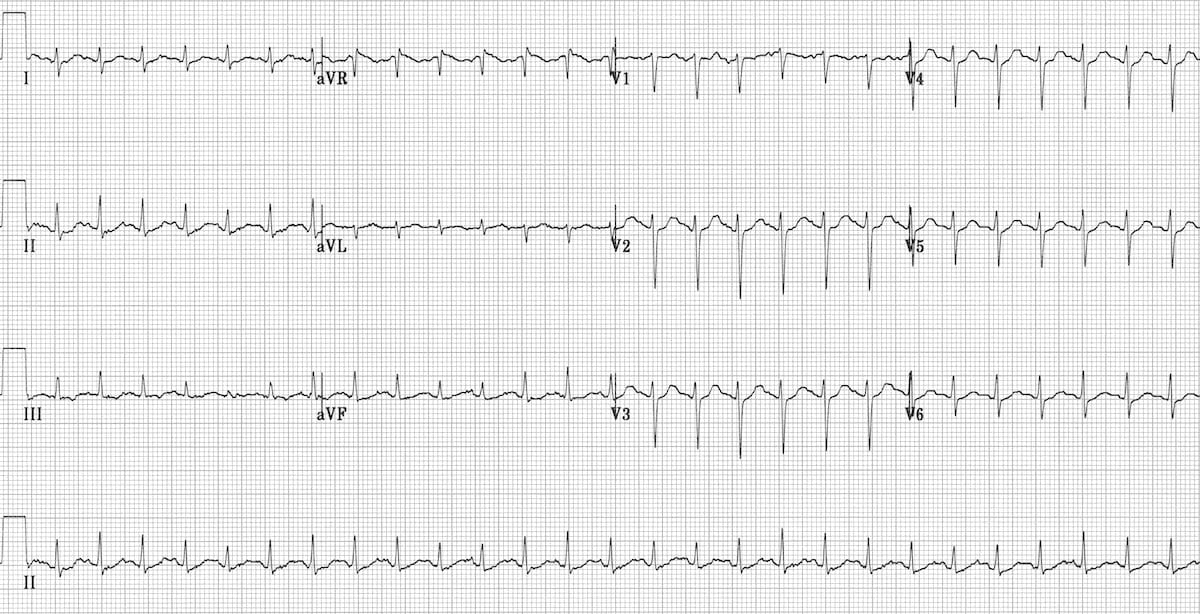


Image by litfl, retrieved from: <https://litfl.com/sinus-tachycardia-ecg-library/> on 2/1/2024. Creative Commons License associated: <https://creativecommons.org/licenses/by-nc-sa/4.0/>.
