# Supplementary material for: Shock Simulation Day: Medical Decision-Making and Communication Skills for Managing a Hypotensive Adult in a Rapid Response
Source: MedEdPORTAL. 2024 Aug 16;20:11430. doi: 10.15766/mep_2374-8265.11430 (PMC11327352; doi:10.15766/mep_2374-8265.11430)
Supplement: Supplementary file 1 — Rapid Response Variceal Bleed Video.mp4Case 1 Critical Action Checklist.docxCase 2 Critical Action Checklist.docxShock Chalk Talk.docxShock Chalk Talk Instructions.docxCase 1 Patient Sign-out.docxCase 2 Patient Sign-out.docxCase 1 Facilitator Guide.docxCase 2 Facilitator Guide.docxCase 1 Supplemental Data.docxCase 2 Supplemental Data.docxDebrief Guide.docxShock Presimulation Survey.docxShock Postsimulation Survey.docx [file mep_2374-8265.11430-s001.zip › L. Debrief Guide.docx]

**Appendix L. Debrief Guide**

Before each simulation, orient the observing participants to the debrief discussion's structure. The debrief will start with a discussion of the communication, leadership, and teamwork dynamics observed during the simulation, followed by a discussion of the medical decision-making. The observers have a significant role in giving their near-peer relationship with the active participants as well as their ‘birds eye view’ of the simulation.

**Overall: 10 minutes**

1. **Frame the debrief:**
   - *Thank you to our rapid response team and leader! To debrief this simulation, we’ll focus on two aspects of leading a rapid response: The team leadership, teamwork, and resource utilization aspect, and then the medical knowledge and management side*
2. **Shared mental model**:
   - *Before we get into the debrief, Could someone summarize what you think was going on with the patient? It’s ok if you don't remember the specifics of the patient, I just want to see if we all on the same page about what was causing them to be altered.*
   - If no responses, ask the rapid response leader
   - Following response with *Does everyone agree with this?*
3. **Teamwork debrief –** this should be the larger portion of the debrief, the chalk talk will review some medical management
   - *We’ll start with the teamwork portion of the simulation.*
   - Some questions to start the discussion – can phrase questions to active participants and observes separately
     - *What do we think went well*
     - *What do we think did not go well?*
   - Points to hit/discuss
     - ***Announce yourself as the rapid response leader***
     - ***Ask for vital signs***
     - ***Assign roles***
     - ***Stand at foot of bed***
     - ***Closed loop communication***- also confirming task completion
     - ***Get help early –*** resident, attending, STAT nurse, MICU, RT etc
     - ***Share your thought process and ask the team for input early-*** create a **shared mental model**
     - *Avoid task saturating nursing staff* – asking for multiple tasks all at once; instead say “I will need A, B, and C. Could you start with A?”
     - *Ask for events leading up to RRT*
     - *Asks for medication list, past medical history, recent labs*
4. **Medical Management debrief –** *What questions do you have about the medical treatment of this patient?*
   - **For learning points to hit if not brought up by students, see each case below**

**Case 1 - Septic Shock**

**Learning points/medical knowledge:** Hit as many of these points as you can, but this will also be discussed in the chalk talk

- Early administration of antibiotics is critical, ideally targeted towards the concerning infectious syndrome, however if a patient is unstable, it is less a time for antibiotic precision and more a time for safety- cover broadly
- Most patients with septic shock should receive 30 ml/kg of fluid resuscitation
- You can start pressors if needed through a peripheral IV before the patient is transferred to the ICU if needed.
- ICU is a level of care, not necessarily a specific location. If the STAT RN is taking care of the patient, they are effectively in an ICU.

**Case 2 - Cardiogenic Shock**

**Learning points/medical knowledge:** Hit as many of these points as you can, but this will also be discussed in the chalk talk

- Have a low threshold to obtain an ECG for a patient showing hemodynamic instability to screen for cardiogenic etiologies
- Nitroglycerin can cause hypotension due to preload dependence in patients with right sided cardiac ischemia
- Load the patient with ASA and start a heparin ggt
- If hemodynamic support is needed, consider a dobutamine trial
- Patients in cardiogenic shock should be transferred to the CCU and cardiology should be called immediately
