# Supplementary material for: Shock Simulation Day: Medical Decision-Making and Communication Skills for Managing a Hypotensive Adult in a Rapid Response
Source: MedEdPORTAL. 2024 Aug 16;20:11430. doi: 10.15766/mep_2374-8265.11430 (PMC11327352; doi:10.15766/mep_2374-8265.11430)
Supplement: Supplementary file 1 — Rapid Response Variceal Bleed Video.mp4Case 1 Critical Action Checklist.docxCase 2 Critical Action Checklist.docxShock Chalk Talk.docxShock Chalk Talk Instructions.docxCase 1 Patient Sign-out.docxCase 2 Patient Sign-out.docxCase 1 Facilitator Guide.docxCase 2 Facilitator Guide.docxCase 1 Supplemental Data.docxCase 2 Supplemental Data.docxDebrief Guide.docxShock Presimulation Survey.docxShock Postsimulation Survey.docx [file mep_2374-8265.11430-s001.zip › K. Case 2 Supplemental Data.docx]

**Appendix K Instructions:** Please pace return of labs, imaging, and diagnostics according to when ordered in the scenario (e.g. if ordered early, delay a bit to encourage interventions in the down time and, if ordered late, may return more rapidly to expedite the scenario completion).

| **NEW DIAGNOSTIC DATA (return if requested ordered)** |  |
| --- | --- |
| **Point-of-care glucose** | 132 mg/dL |
| **Complete blood count** | WBC 7.2 K/uL, Hct 39%, Plts 257 K/uL |
| **Basic Metabolic Panel** | Na 129 mEq/L, K 3.9 mEq/L, Cl 96 mEq/L, HCO3 13 mEq/L, BUN 42 mg/dL, Cr 1.3 mg/dL, Ca 9.4 mg/dL, Mg 1.7 mg/dL |
| **Liver Function Tests** | AST 31 U/L, ALT 42 U/L, Alkaline Phosphatase 114 U/L, Total Bilirubin 0.5 mg/dL |
| **Arterial Blood Gass** | pH 7.31, pCO2 34 mmHg, pO2 89 mmHg, HCO3 13 mEq/L |
| **Lactate** | 1.8 mmol/L |
| **Troponin** | 1.026 ng/mL |
| **Coagulation studies** | PTT 34.1 secs, PT 14.1 secs, INR 0.9 |

**Chest Radiograph**


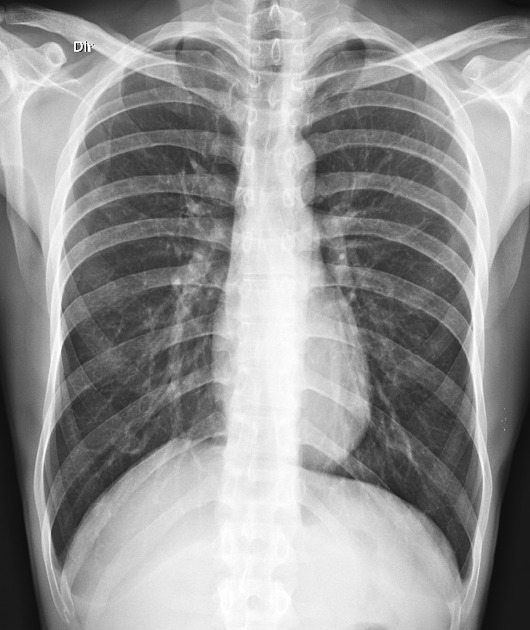


Case courtesy of Bruno Di Muzio, Radiopaedia.org, rID: 37906. Image retrieved from <https://radiopaedia.org/cases/normal-frontal-chest-x-ray-1?lang=us> on 3/8/2024.

**Electrocardiogram**


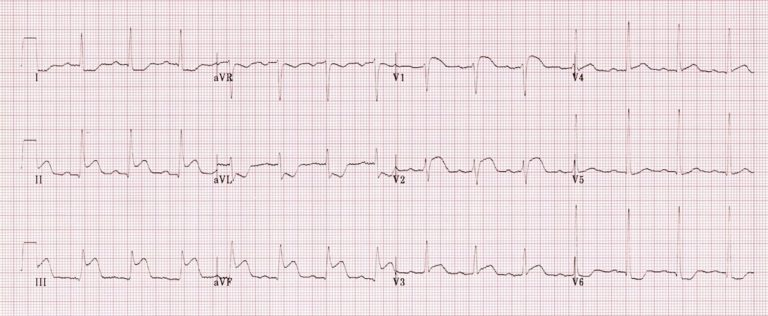


Image by litfl, retrieved from: <https://litfl.com/right-ventricular-infarction-ecg-library/> on 2/1/2024. Creative Commons License associated: https://creativecommons.org/licenses/by-nc-sa/4.0/.

**Electrocardiogram with lead 4 in the right position, provide only if asked specifically by learners.**


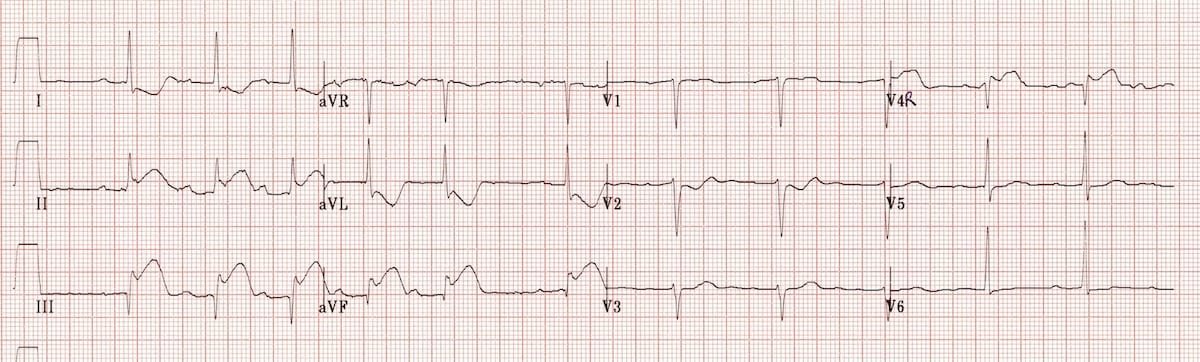


Image by litfl, retrieved from: <https://litfl.com/right-ventricular-infarction-ecg-library/> on 2/1/2024. Creative Commons License associated: https://creativecommons.org/licenses/by-nc-sa/4.0/.
