# Supplementary material for: Shock Simulation Day: Medical Decision-Making and Communication Skills for Managing a Hypotensive Adult in a Rapid Response
Source: MedEdPORTAL. 2024 Aug 16;20:11430. doi: 10.15766/mep_2374-8265.11430 (PMC11327352; doi:10.15766/mep_2374-8265.11430)
Supplement: Supplementary file 1 — Rapid Response Variceal Bleed Video.mp4Case 1 Critical Action Checklist.docxCase 2 Critical Action Checklist.docxShock Chalk Talk.docxShock Chalk Talk Instructions.docxCase 1 Patient Sign-out.docxCase 2 Patient Sign-out.docxCase 1 Facilitator Guide.docxCase 2 Facilitator Guide.docxCase 1 Supplemental Data.docxCase 2 Supplemental Data.docxDebrief Guide.docxShock Presimulation Survey.docxShock Postsimulation Survey.docx [file mep_2374-8265.11430-s001.zip › D. Shock Chalk Talk.docx]

**Appendix D. Shock Chalk Talk**

| **Primary Survey** | **Framework** | **Preload** | **CO** | **Afterload** | **JVP** | **Pulmonary Edema** | **Extremities** | **Differential** | **Initial Managment** |
| --- | --- | --- | --- | --- | --- | --- | --- | --- | --- |
| Full set of vitals  Focused Physical Exam  Cardiopulmonary  JVP  Extremities  **Mental Status**  **Call for help!** | Hypovolemic |  |  |  |  | No | Cool | Volume loss, Dehydration, Hemorrhage | Volume:  Crystalloid vs Blood |
|  | Cardiogenic |  |  |  |  | Yes | Cool | Heart failure, Valvular, Arrythmia | Inotropes +/- diuresis. Antiarrhythmics |
|  | Distributive | /= | /= |  | **=** | Yes/No | Warm | Sepsis, anaphylaxis, neurogenic, adrenal insuff. | IVF +/- Norepi/ Vaso |
|  | Obstructive |  |  |  |  | No | Cool | PE, Tension peumo, Cardiac Tamponade | Relieve obstruction +/- Inotropes |
