# Supplementary material for: Shock Simulation Day: Medical Decision-Making and Communication Skills for Managing a Hypotensive Adult in a Rapid Response
Source: MedEdPORTAL. 2024 Aug 16;20:11430. doi: 10.15766/mep_2374-8265.11430 (PMC11327352; doi:10.15766/mep_2374-8265.11430)
Supplement: Supplementary file 1 — Rapid Response Variceal Bleed Video.mp4Case 1 Critical Action Checklist.docxCase 2 Critical Action Checklist.docxShock Chalk Talk.docxShock Chalk Talk Instructions.docxCase 1 Patient Sign-out.docxCase 2 Patient Sign-out.docxCase 1 Facilitator Guide.docxCase 2 Facilitator Guide.docxCase 1 Supplemental Data.docxCase 2 Supplemental Data.docxDebrief Guide.docxShock Presimulation Survey.docxShock Postsimulation Survey.docx [file mep_2374-8265.11430-s001.zip › E. Shock Chalk Talk Instructions.docx]

**Appendix E. Shock Chalk Talk Instructions**

**Learning Objectives:**

**By the end of the chalk talk, learners will be able to:**

1) Describe a framework for the primary survey for a patient who is in shock.
2) Differentiate the four primary categories of shock by physiology and physical exam characteristics.
3) Determine the initial steps in management for the four primary types of shock physiology.

1. **Primary Survey:**

**You have been called to a patient's room because the nurse has informed you that the patient is hypotensive to 70s/40s. What is the first piece of information that you need to ask for from the nurse?** Full set of vitals.

*Emphasize the importance of getting a complete picture of vitals immediately as this will impact your initial management and triage of the clinical scenario.*

**What are you going to specifically look for when you examine the patient?** JVP, extremities, cardiovascular, respiratory, and mental status.

*Have the learners list the high yield portions of the physical exam that they would target. The focused physical exam in a rapid response should take 1-2 minutes. Highlight the importance of mental status in triaging the clinical severity of the patient's hypotension.*

**You've completed you exam and now its you, the nurse, and the patient in the room. Is there anybody else that you want to call now?**

*Answers can vary (RT, STAT RN, MICU Team, anesthesia). Stress that calling for help early is always preferred. If there is any concern for an inability to protect their airway, calling anesthesia should always be prioritized early.*

**B. Shock Framework**

Have the learners fill out the shock framework as a group. Complete the physiology section for all four shock types first. Then complete the physical exam section. The differential diagnosis and initial management for each subtype of shock can be filled out together.

**C. Take home points**

1. Always obtain a complete set of vitals when evaluating an unstable patient
2. Practice your focused physical exam to get the information you need in 1 minute
3. Call for help early
4. Focus on key physical exam findings of JVP, pulmonary edema, and extremities to help you differentiate the 4 types of shock.
