# Supplementary material for: Shock Simulation Day: Medical Decision-Making and Communication Skills for Managing a Hypotensive Adult in a Rapid Response
Source: MedEdPORTAL. 2024 Aug 16;20:11430. doi: 10.15766/mep_2374-8265.11430 (PMC11327352; doi:10.15766/mep_2374-8265.11430)
Supplement: Supplementary file 1 — Rapid Response Variceal Bleed Video.mp4Case 1 Critical Action Checklist.docxCase 2 Critical Action Checklist.docxShock Chalk Talk.docxShock Chalk Talk Instructions.docxCase 1 Patient Sign-out.docxCase 2 Patient Sign-out.docxCase 1 Facilitator Guide.docxCase 2 Facilitator Guide.docxCase 1 Supplemental Data.docxCase 2 Supplemental Data.docxDebrief Guide.docxShock Presimulation Survey.docxShock Postsimulation Survey.docx [file mep_2374-8265.11430-s001.zip › F. Case 1 Patient Sign-out.docx]

**Appendix F. Instructions:** Provide patient hand-off information to learners before simulation case begins.

| **FLOOR – 6NE** | **VITALS-LABS** | **Ins/Outs – Cultures** | **MEDS** | **COMMENTS/TASKS** |
| --- | --- | --- | --- | --- |
| Ranka, Justin  2345  DOB: 9/15/63 59 M  6202-01  **Code: Full Code**  **Allergy:**  NKDA  Prob/Proc   - DMT2   Hypertension   - Gout - Nephrolithiasis | T 36.8 p82 NIBP 124/78 Art/ r18 sat 97% RA  WBC 8.3 K/uL,  Hct 35 %,  Plts 315 K/uL  Na 132 mEq/L,  K 4.0 mEq/L,  Cl 97 mEq/L,  HCO3 15 mEq/L,  BUN 32 mg/dL,  Cr 1.1 mg/dL,  Glucose 155 mg/dL |  | Allopurinol 100 mg Daily  Amlodipine 5 mg daily  Insulin glargine 5 units QHS  Tamsulosin 0.4 mg  Oxycodone 5-10 mg q4h  PRN  Senna 17.2 mg daily  Enoxaparin 40 mg QHS | Stable/Full code  59 y/o man with DMT2, HTN, gout presented last night with abd pain found to have L ureteral stone w/ hydro s/p lithotripsy today. Tolerated well, no complications, plan to dc in AM.  Today:  -lithotripsy today, no issues  -Ordered a diet  -wife updated  -Glucose 160-210 mg/dL  #Nephrolithiasis  #DMT2; insulin  XC: NTD |
