# Supplementary material for: Shock Simulation Day: Medical Decision-Making and Communication Skills for Managing a Hypotensive Adult in a Rapid Response
Source: MedEdPORTAL. 2024 Aug 16;20:11430. doi: 10.15766/mep_2374-8265.11430 (PMC11327352; doi:10.15766/mep_2374-8265.11430)
Supplement: Supplementary file 1 — Rapid Response Variceal Bleed Video.mp4Case 1 Critical Action Checklist.docxCase 2 Critical Action Checklist.docxShock Chalk Talk.docxShock Chalk Talk Instructions.docxCase 1 Patient Sign-out.docxCase 2 Patient Sign-out.docxCase 1 Facilitator Guide.docxCase 2 Facilitator Guide.docxCase 1 Supplemental Data.docxCase 2 Supplemental Data.docxDebrief Guide.docxShock Presimulation Survey.docxShock Postsimulation Survey.docx [file mep_2374-8265.11430-s001.zip › G. Case 2 Patient Sign-out.docx]

**Appendix G.** **Instructions:** Provide patient hand-off information to learners before simulation case begins.

| **FLOOR – 6NE** | **VITALS-LABS** | **Ins/Outs – Cultures** | **MEDS** | **COMMENTS/TASKS** |
| --- | --- | --- | --- | --- |
| BLACK, DEBORAH  1234  DOB: 2/15/59, 72 F  6234-01  **Code: Full Code**  **Allergy:** NKDA  Prob/Proc   - DMT2 - Hyperglycemia - Hypertension - Hyperlipidemia - P Afib - GLF | T 36.5 p78 NIBP 124/74 Art/ r18 sat 98% on RA  WBC 5.1 K/uL,  Hct 31 %,  Plts 273 K/uL  Na 136 mEq/L,  K 3.7 mEq/L,  Cl 99 mEq/L,  HCO3 19 mEq/L,  BUN 21 mg/dL,  Cr 0.7 mg/dL,  Glucose 172 mg/dL | **-** | SCH MEDS  Lisinopril 20 mg daily  Atorvastatin 40 mg daily  Metoprolol succinate 75 mg daily  Apixaban 5 mg BID  Melatonin 3 mg daily  Cholecalciferol 600 units daily  Senna 17.2 mg daily  Polyethylene glycol 17 grams daily PRN  PRN  Acetaminophen 650 mg q6h PRN | Stable/Full code  72 y/o F with PMH of A fib, HTN, HLD, and DMT2 p/w GLF. Work up negative plan for dc in AM.  Today:  Worked with PT/OT. No arrythmia on tele. Orthostatic negative, likely dc in AM after TTE is obtained  XC: NTD |
