# Supplementary material for: Shock Simulation Day: Medical Decision-Making and Communication Skills for Managing a Hypotensive Adult in a Rapid Response
Source: MedEdPORTAL. 2024 Aug 16;20:11430. doi: 10.15766/mep_2374-8265.11430 (PMC11327352; doi:10.15766/mep_2374-8265.11430)
Supplement: Supplementary file 1 — Rapid Response Variceal Bleed Video.mp4Case 1 Critical Action Checklist.docxCase 2 Critical Action Checklist.docxShock Chalk Talk.docxShock Chalk Talk Instructions.docxCase 1 Patient Sign-out.docxCase 2 Patient Sign-out.docxCase 1 Facilitator Guide.docxCase 2 Facilitator Guide.docxCase 1 Supplemental Data.docxCase 2 Supplemental Data.docxDebrief Guide.docxShock Presimulation Survey.docxShock Postsimulation Survey.docx [file mep_2374-8265.11430-s001.zip › M. Shock Presimulation Survey.docx]

**Appendix M. Shock Presimulation Survey**

This information will be used only for program improvement, it is not intended as an individual evaluation. Your name will only be used to link your pre and post simulation surveys.

1. **How many rapid responses have you been a part of during your intern year?**

**0**

**1**

**2-3**

**4+**

1. **Did you watch the hemorrhagic shock (variceal bleed) rapid response video module prior to today?**

**Yes**

**No**

1. **Have you attended another rapid response simulation? If so what was the topic?**

**Yes; Topic: ___________________________________________**

**No**

1. **Rate your level of confidence in leading a rapid response:**

| Not at all confident | Not very confident | Neutral | Somewhat confident | Extremely confident |
| --- | --- | --- | --- | --- |
|  |  |  |  |  |

1. **Rate your level of confidence to manage the following emergencies during a rapid response:**

|  | Not at all confident | Not very confident | Neutral | Somewhat confident | Extremely confident |
| --- | --- | --- | --- | --- | --- |
| Undifferentiated hypotension |  |  |  |  |  |
|  | Not at all confident | Not very confident | Neutral | Somewhat confident | Extremely confident |
| Septic shock |  |  |  |  |  |
| Cardiogenic shock |  |  |  |  |  |
| Hemorrhagic shock |  |  |  |  |  |
| Anaphylactic shock |  |  |  |  |  |

**Knowledge:**

1. **What labs should you order for a patient with sepsis?**

1. **How much IV fluid is recommended in most patients for the treatment of septic shock?**
2. **What concerns do you have regarding leading a rapid response?**

**Medical knowledge**

**Team leadership**

**Resource utilization**

**Communication in a high stress environment**

**Other**
