# Supplementary material for: Shock Simulation Day: Medical Decision-Making and Communication Skills for Managing a Hypotensive Adult in a Rapid Response
Source: MedEdPORTAL. 2024 Aug 16;20:11430. doi: 10.15766/mep_2374-8265.11430 (PMC11327352; doi:10.15766/mep_2374-8265.11430)
Supplement: Supplementary file 1 — Rapid Response Variceal Bleed Video.mp4Case 1 Critical Action Checklist.docxCase 2 Critical Action Checklist.docxShock Chalk Talk.docxShock Chalk Talk Instructions.docxCase 1 Patient Sign-out.docxCase 2 Patient Sign-out.docxCase 1 Facilitator Guide.docxCase 2 Facilitator Guide.docxCase 1 Supplemental Data.docxCase 2 Supplemental Data.docxDebrief Guide.docxShock Presimulation Survey.docxShock Postsimulation Survey.docx [file mep_2374-8265.11430-s001.zip › N. Shock Postsimulation Survey.docx]

**Appendix N. Shock Postsimulation Survey**

This information will be used only for program improvement, it is not intended as an individual evaluation. Your name will only be used to link your pre and post simulation surveys.

1. **What role did you play in the sepsis simulation today?**
   1. **Rapid response leader**
   2. **Intern**
   3. **Observed the simulation**
2. **What role did you play in the cardiogenic shock simulation today?**
   1. **Rapid response leader**
   2. **Intern**
   3. **Observed the simulation**
3. **Do you feel that your skills and knowledge in leading a rapid response improved today?**

**Yes**

**No**

1. **Do you feel that your confidence in leading a rapid response improved today?**

**Yes**

**No**

1. **Rate your level of confidence in leading a rapid response:**

| Not at all confident | Not very confident | Neutral | Somewhat confident | Extremely confident |
| --- | --- | --- | --- | --- |
|  |  |  |  |  |

1. **Do you feel that your confidence to manage shock improved today?**

**Yes**

**No**

1. **Rate your level of confidence to manage the following emergencies during an RRT**

|  | Not at all confident | Not very confident | Neutral | Somewhat confident | Extremely confident |
| --- | --- | --- | --- | --- | --- |
| Undifferentiated hypotension |  |  |  |  |  |
|  | Not at all confident | Not very confident | Neutral | Somewhat confident | Extremely confident |
| Septic shock |  |  |  |  |  |
| Cardiogenic shock |  |  |  |  |  |
| Hemorrhagic shock |  |  |  |  |  |
| Anaphylactic shock |  |  |  |  |  |

1. **What labs should you order for a patient with sepsis?**

1. **How much IV fluid is recommended in most patients to treat septic shock?**
2. **What concerns do you have after today’s session regarding leading a rapid response?**

**Medical knowledge**

**Team leadership**

**Resource utilization**

**Communication in a high stress environment**

**Other**

1. **Did you find this simulation session helpful?**

**Yes**

**No**

1. **Would you want to attend more of these simulation sessions?**

**Yes**

**No**

1. **Do you think this session will improve your management of a patient with shock?**

**Yes**

**No**

1. **Do you think this session helped to improve your team leadership skills?**

**Yes**

**No**

1. **When do you think would have been the best time to have this simulation training?**

**During Orientation**

**Summer of Intern year**

**Now- it was good timing to have this training**

**End of Intern year**

**Sometime second year or later**

1. **Please rate the teaching effectiveness of the following instructor(s):**

| Faculty | Not at all effective | Not very effective | Neutral | Somewhat effective | Extremely effective |
| --- | --- | --- | --- | --- | --- |
|  |  |  |  |  |  |
|  |  |  |  |  |  |

1. **Please provide any other feedback that you have:**
